# Pilgrims and MERS-CoV: what’s the risk?

**DOI:** 10.1186/s12982-015-0025-8

**Published:** 2015-02-18

**Authors:** Tarek Soliman, Alex R Cook, Richard J Coker

**Affiliations:** Saw Swee Hock School of Public Health, National University of Singapore and National University Health System, Tahir Foundation Building, 12 Science Drive 2, Singapore, 117549 Republic of Singapore; Yale-NUS College, 6 College Ave E, Singapore, 138614 Republic of Singapore; London School of Hygiene and Tropical Medicine, Keppel St, Bloomsbury, London, WC1E 7HT UK

**Keywords:** Coronavirus, Mass gathering, Kingdom of Saudi Arabia

## Abstract

The risk of Middle East Respiratory Syndrome Coronavirus spreading globally is worrying, given the annual mass gathering of the Hajj and the year-long influx of pilgrims undertaking the Umrah. Based on the incidence in Saudi Arabia since June 2012, the most likely scenario given recent pilgrim numbers is estimated to be one case per Hajj, and three Umrah pilgrims per year, but which could plausibly reach seven and ten pilgrims respectively. In addition to the 2015 Hajj, national surveillance systems should be on the alert for the low but long-lasting risk of infected pilgrims returning from the Umrah throughout the year.

## Background

A decade after SARS, another coronavirus associated with high mortality rates is the subject of global concern [1]. As of 21st of December 2014, there have been 938 cases of Middle East Respiratory Syndrome Coronavirus (MERS-CoV), the vast majority of which (822) have been identified within the Kingdom of Saudi Arabia (KSA) [2]. Of the Saudi cases, 355 (43%) have died [2], prompting the World Health Assembly to express its concern about the situation [3]. The risk of international disease spread is especially worrying given KSA’s role as the home of the most important pilgrimage sites in Islam, which leads to the large mass gathering of the annual Hajj, in which two million pilgrims converge on the KSA, and the ‘minor’ Umrah, which sees six million pilgrims arrive throughout the rest of the year. During the 2013 Hajj, Saudi authorities did not report any MERS-CoV cases, although later, findings have been reported in several countries such as the United Kingdom, Malaysia, Jordan and the United Arab Emirates [4]. However, given the recent upsurge in cases in the KSA that began in April 2014 [5], and detected cases among returning pilgrims from the Umrah [6], the risk from returning pilgrims should not be ignored. In particular, countries with sizeable Muslim populations need to prepare, not just during the next Hajj, but for cases among pilgrims returning from the year-round Umrah. Understanding what the risk to pilgrims is, what the risks of pilgrims returning with MERS-CoV is, and whether human to human transmission on their return poses a public health threat, is vital. Surveillance and control measures in response to this threat may need to be adapted or developed.

## Methods

The risk of infection to pilgrims coming from different regions can be estimated based on overall incidence of disease in humans in the KSA of MERS-CoV since the first infection in June 2012. Each year, the number of pilgrims entering KSA for the Hajj and Umrah is determined by a quota set by the Saudi Authorities, based upon the total country size and the proportion of the country that is Muslim [7]. This quota was used to develop a spatial distribution of risk by region of the world (Table 1). As MERS-CoV infection is a rare event, the risks of at least one infection in the travellers from each region were determined from the Poisson distribution parameterised by the expected number of cases during the Hajj period and throughout the year for Umrah [8]. The number of cases in which countries in each region should prepare for can be represented by the 95 percent upper bound of the distribution. The expected number of cases is calculated by multiplying the number of pilgrims entering KSA [9], the average duration of time pilgrims stay in KSA (which is around 21 days for Hajj and 7 days for Umrah) [10], and the incidence of MERS-CoV per day, estimated based on reports from KSA [2].

**Table 1:** **Potential risk of MERS-CoV during the Hajj and Umrah**

**Region**	**Pilgrimage**	**Number of Pilgrims, thousands** ^**1**^	**Risk of at least one case (%)**	**Expected number of pilgrims returning with MERS-CoV** ^**2**^	**Max cases (95% uncertainty)**
Southeast Asia	Hajj	201	10%	0	1
	Umrah	577	11%	0	1
Central and North Asia	Hajj	206	10%	0	1
	Umrah	594	12%	0	1
South Asia	Hajj	435	21%	0	1
	Umrah	1,252	21%	1	2
East Asia	Hajj	20	1%	0	0
	Umrah	59	1%	0	0
North Africa	Hajj	167	9%	0	1
	Umrah	482	10%	0	1
Middle East	Hajj	805	35%	1	2
	Umrah	2,315	31%	1	3
Sub-Saharan Africa	Hajj	208	11%	0	1
	Umrah	599	12%	0	1
Europe	Hajj	38	2%	0	0
	Umrah	109	2%	0	1
Americas	Hajj	5	~0%	0	0
	Umrah	13	~0%	0	0
**Total** ^**3**^	Hajj	2,085	100%	1	7
	Umrah	6,000	100%	3	10

^1^Estimated values based on the ‘proportional rule’ of Hajj. The Middle East figure accounts for internal Saudi pilgrims, for whom the proportional rule differs.

^2^Numbers of cases are rounded.

^3^Totals of the ‘expected number of Umrah pilgrims returning with MERS-CoV’ differs from the summation of the expected number per region due to rounding.

## Results

Based on the incidence in the KSA since June 2012, we estimate that the most likely scenario is of one pilgrim becoming infected, with an estimated maximum of seven cases, during each period of the Hajj. If there are infections, pilgrims from the Middle East, including pilgrims originating in the KSA, would constitute around 35 percent of cases, markedly lower than their share of infections thus far (89 percent) [5]. The risk of at least one case in the Middle East was estimated to be 54 percent. Other areas at potential risk are Africa and most of Asia, given their large Muslim populations. The proportion of cases from (Central and North, South, and Southeast) Asia and Africa were estimated at 41 and 18 percent respectively. By contrast, the Americas, Europe and East Asia are likely to witness few if any cases in returning pilgrims. Best estimates are that three cases are likely to occur during the Umrah, with a maximum of ten cases (Table 1).

## Conclusion

Despite the circulation of MERS-CoV in the KSA since 2012, and conditions of overcrowding, and intense human-to-human contacts during the Hajj in particular, there has not been the rapid dissemination of MERS-CoV among returning pilgrims that might have been feared. Given this, and the lack of onward transmission from those pilgrims who subsequently were reported to be infected suggests that MERS-CoV does not pose a global threat of the same level as did Severe Acute Respiratory Syndrome (SARS). As the incubation period has been estimated to be shorter than the total length of stay of Hajj pilgrims and longer than the length of stay of Umrah pilgrims in KSA, it is likely that most infections during the Hajj will have onset while still in KSA, while any infected Umrah cases are more likely to present in their home countries. In principle, our estimate of a small number of pilgrims likely to be infected during the Hajj and Umrah could be an underestimate, if overcrowding and the arduous physical demands that pilgrims experience play a part in transmission and disease progression [10], however, the many opportunities for transmission over several years supports our low estimates. Epidemiological analysis of infected cases does not support human to human transmission, although uncertainty is still present in the sources of infection and possible transmission modes. As a result, port and national surveillance systems as well as clinicians and travel medical teams should be on the alert for the risk of small number of MERS-CoV returning from KSA, not just during the next Hajj, but less-obviously throughout the year from the Umrah. Shortage in resource capacities and inequitably distributed medical resources in some potential low and middle income countries in Africa and Asia may call for additional international assistance to prevent and control infection. A timely mobilization of essential resources could potentially prevent further dispersal of the disease in these countries.

### Ethics committee approval

There was no need to obtain ethics committee approval.
